# An experimental medicine decipher of a minimum correlate of cellular immunity: Study protocol for a double-blind randomized controlled trial

**DOI:** 10.3389/fimmu.2023.1135979

**Published:** 2023-03-10

**Authors:** Shirin Kalimuddin, Yvonne F. Z. Chan, October M. Sessions, Kuan Rong Chan, Eugenia Z. Ong, Jenny G. Low, Antonio Bertoletti, Eng Eong Ooi

**Affiliations:** ^1^ Department of Infectious Diseases, Singapore General Hospital, Singapore, Singapore; ^2^ Duke-NUS Medical School, Singapore, Singapore; ^3^ Saw Swee Hock School of Public Health, National University of Singapore, Singapore, Singapore; ^4^ Department of Pharmacy, National University of Singapore, Singapore, Singapore; ^5^ Viral Research and Experimental Medicine Centre (ViREMiCS), SingHealth Duke-NUS Academic Medical Centre, Singapore, Singapore; ^6^ Singapore Immunology Network, Agency for Science, Technology and Research (ASTAR) Singapore, Singapore, Singapore

**Keywords:** yellow fever, Japanese Encephalitis, vaccine, cellular immunity, T-cells

## Abstract

Vaccination induces an adaptive immune response that protects against infectious diseases. A defined magnitude of adaptive immune response that correlates with protection from the disease of interest, or correlates of protection (CoP), is useful for guiding vaccine development. Despite mounting evidence for the protective role of cellular immunity against viral diseases, studies on CoP have almost exclusively focused on humoral immune responses. Moreover, although studies have measured cellular immunity following vaccination, no study has defined if a “threshold” of T cells, both in frequency and functionality, is needed to reduce infection burden. We will thus conduct a double-blind, randomized clinical trial in 56 healthy adult volunteers, using the licensed live-attenuated yellow fever (YF17D) and chimeric Japanese encephalitis-YF17D (JE-YF17D) vaccines. These vaccines share the entire non-structural and capsid proteome where the majority of the T cell epitopes reside. The neutralizing antibody epitopes, in contrast, are found on the structural proteins which are not shared between the two vaccines and are thus distinct from one another. Study participants will receive JE-YF17D vaccination followed by YF17D challenge, or YF17D vaccination followed by JE-YF17D challenge. A separate cohort of 14 healthy adults will receive the inactivated Japanese Encephalitis virus (JEV) vaccine followed by YF17D challenge that controls for the effect of cross-reactive flaviviral antibodies. We hypothesize that a strong T cell response induced by YF17D vaccination will reduce JE-YF17D RNAemia upon challenge, as compared to JE-YF17D vaccination followed by YF17D challenge. The expected gradient of YF17D-specific T cell abundance and functionality would also allow us to gain insight into a T cell threshold for controlling acute viral infections. The knowledge gleaned from this study could guide the assessment of cellular immunity and vaccine development.

**Clinical trial registration:**

Clinicaltrials.gov, NCT05568953.

## Introduction

Outbreaks of emerging and re-emerging viruses from animal reservoirs continue to plague mankind. In the past 20 years alone, at least eight viruses have emerged from zoonotic sources to cause epidemics and pandemics, the majority of which have no effective vaccines. Vaccine development is a costly endeavor, with many failed candidates that never achieve licensure. As clearly evidenced by the coronavirus disease 2019 (COVID-19) pandemic, the public health and socio-economic impact of viral outbreaks can be devastating, but can be dramatically reversed with mass vaccination. This further emphasizes the critical need to develop safe and effective viral vaccines in a timely and cost-effective manner.

The selection of vaccine candidates for clinical use is best guided by well-defined correlates of protection (CoP), i.e. an immune response that is responsible for, even if only in part, and statistically interrelated with protection (1). To date, vaccine development has relied heavily on measuring vaccine-induced antibody levels, a component of humoral immunity, as a correlate of protection. Humoral immunity, however, is only one arm of the overall host immune response to infection (2). Viral infection also elicits a cellular immune response, driven primarily by helper and cytotoxic T cells (3). Virus-specific memory T cells that develop following infection or immunization can be rapidly recalled during re-infection to aid antibody production (helper CD4+ T cells), and directly kill virus-infected cells (cytotoxic CD8+ T cells) (4, 5). Thus, while antibodies can prevent infection, T cells play an equally important role in reducing the total viral burden that drives inflammation and severe disease pathogenesis.

Indeed, the importance and protective role of T cell immunity has been clearly demonstrated in the context of flaviviral infections. Stronger and more polyfunctional antigen-specific T cell responses have been found in individuals with HLA alleles associated with a reduced susceptibility to severe dengue (6, 7), while asymptomatic dengue virus (DENV) infection has been associated with higher levels of cytokine-secreting T cells compared to symptomatic infection (8). The importance of T cell immunity has also been demonstrated in the context of dengue vaccine development. CYD-TDV, the first dengue vaccine to be licensed for use, is a live-attenuated vaccine comprising the structural pre-membrane and envelope genes of the four dengue serotypes with the non-structural and capsid genes of the live-attenuated yellow fever vaccine YF17D. Most of the CD8+ epitopes reside on the DENV non-structural proteins (9), which CYD-TDV does not contain, resulting in a limited DENV-specific T cell response. This likely contributed to the lacklustre efficacy observed with CYD-TDV in phase III trials, especially against DENV-1 and -2, despite robust levels of neutralizing antibodies (10, 11). In contrast, YF17D vaccine elicits a broad and polyfunctional yellow-fever specific T-cell response, demonstrating excellent efficacy against yellow fever, with a single dose conferring lifelong immunity (12).

Moving forward, it is thus crucial that future vaccine development efforts take into account the importance of T cell immunity in determining vaccine efficacy, and not focus solely on neutralizing antibody levels as a correlate of protection, as is currently often currently the case. Defining a minimum threshold of T-cell immunity, both in terms of frequency and functionality, would thus be critical in order to develop and select effective vaccines for the future.

## Hypothesis and objectives

**Figure 1:**
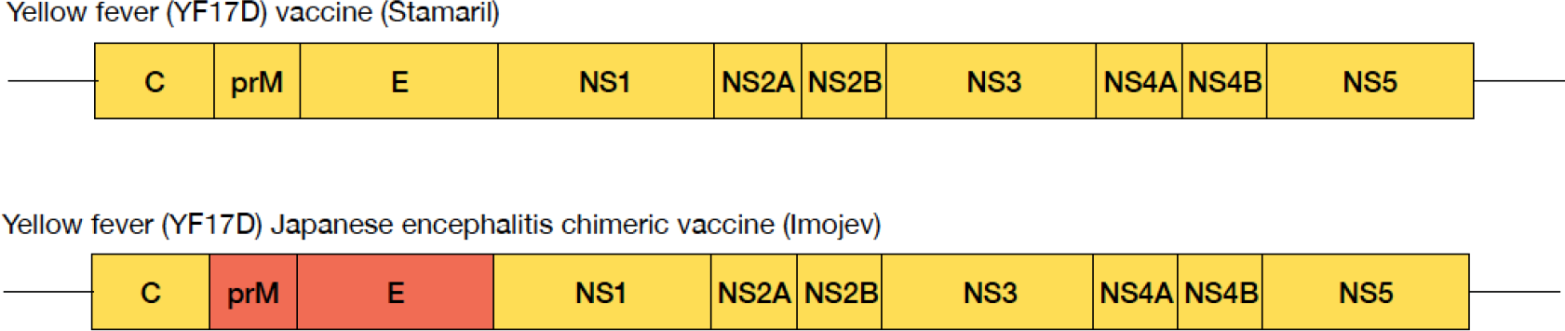
Schematic representation of the genome of YF17D and chimeric JE-YF17D vaccines. JE virus prM and E genes (shown in red) have replaced those of YF17D in the chimeric virus.

The overall goal of this study is to define a minimum quantitative threshold of T cells induced by vaccination needed to control subsequent infection, independent of antibodies. We will conduct an experimental study in healthy adult volunteers, leveraging on the immunogenic properties of the licensed live-attenuated yellow fever (YF17D; STAMARIL^®^, Sanofi Pasteur) and chimeric Japanese encephalitis-YF17D (JE-YF17D; IMOJEV^®^, Sanofi Pasteur) vaccines. These vaccines contain the same genomic YF17D backbone (13, 14): vaccination with either vaccine would likely produce T cell responses that, like other flaviviruses, are preferentially directed against the non-structural proteins encoded in their shared genomic backbone, and hence would cross-protect against each other (**Figure 1**). YF17D and JE-YF17D, however, encode different structural pre-membrane (prM) and envelope (E) proteins: vaccination with either would produce neutralizing antibodies protective only against that virus (15). Moreover, YF17D and JE-YF17D vaccination induce different levels of infection as determined by vaccine RNAemia (16, 17), and expectedly differing levels of innate immune responses, allowing us to evaluate how RNAemia and innate immune profile shapes T cell response. The properties of these two live-attenuated vaccines thus provide a unique opportunity to differentiate the role of T cell immunity from humoral immunity in controlling viral infection. In addition, we will also use the licensed inactivated Japanese Encephalitis virus (JEV; IXIARO^®^, Valneva) vaccine to control for the effects of cross-reactive anti-flaviviral antibodies, although we have not observed suppression of YF17D RNAemia from inactivated JEV vaccination (16). Overall, we hypothesize that a high CD4+ and CD8+ T cell response will reduce RNAemia upon challenge with a structurally heterologous virus, and correspondingly result in reduced magnitude of host response to challenge infection. The specific objectives of the study are as follows:

### Primary objective

1. To compare, after challenge with a structurally heterologous vaccine (i.e. JE-YF17D or YF17D), the differences in RNAemia levels (as measured by RT-qPCR) between healthy adults who received primary vaccination with either YF17D vaccine, chimeric JE-YF17D vaccine, or inactivated JEV vaccine.

### Secondary objectives

1. To compare the differences in magnitude and quality of the T cell response induced by primary JE-YF17D, YF17D, and inactivated JEV vaccination in healthy adults.
2. To compare the differences in RNAemia levels after primary vaccination with either YF17D or JE-YF17D in healthy adults.
3. To compare, after challenge with a structurally heterologous vaccine (i.e. JE-YF17D or YF17D), the differences in duration of RNAemia between healthy adults who received primary vaccination with either YF17D vaccine, chimeric JE-YF17D vaccine, or inactivated JEV vaccine.
4. To compare, after challenge with a structurally heterologous vaccine (i.e. JE-YF17D or YF17D), the differences in magnitude and quality of T cell responses between healthy adults who received primary vaccination with either YF17D vaccine, chimeric JE-YF17D vaccine, or inactivated JEV vaccine.
5. To compare, after challenge with a structurally heterologous vaccine (i.e. JE-YF17D or YF17D), the differences in neutralizing antibody titers between healthy adults who received primary vaccination with either YF17D vaccine, chimeric JE-YF17D vaccine, or inactivated JEV vaccine.
6. To compare, after challenge with a structurally heterologous vaccine (i.e. JE-YF17D or YF17D), differences in systemic vaccine-related symptoms between healthy adults who received primary vaccination with either YF17D vaccine, chimeric JE-YF17D vaccine, or inactivated JEV vaccine.

## Methods and analysis

### Trial approval and conduct

The trial is approved by the SingHealth Centralised Institutional Review Board (CIRB) (Ref: 2021/2738) and is registered on clinicaltrials.gov (NCT05568953). The trial sponsor is the Singapore General Hospital, Singapore, in collaboration with Duke-NUS Medical School, Singapore. The trial will be conducted at the SingHealth Investigational Medicine Unit, and carried out in accordance with the principles of the Singapore Good Clinical Practice guidelines and in compliance with the Helsinki Declaration. Written informed consent written will be obtained from all study participants before enrolment.

### Study population

A total of 70 flavivirus-naïve healthy adult volunteers meeting all inclusion criteria and non-exclusion criteria will be enrolled and recruited into the study.

#### Inclusion criteria

1. Healthy adults, male or female, aged between 21 – 45 years at the time of screening.
2. Willingness to comply to study procedures and adhere to study schedule visits.
3. Satisfactory baseline medical assessment as assessed by physical examination and a stable health status. The laboratory values must be within the normal range of the assessing site or show abnormalities that are deemed not clinically significant as judged by the investigator. A stable health status is defined as the absence of a health event satisfying the definition of a serious adverse event.
4. Accessible vein for blood collection.
5. Ability to provide informed consent.
6. Female subjects of non-child bearing potential due to surgical sterilization (hysterectomy or bilateral oophorectomy or tubal ligation) or menopause. Post-menopausal subjects must have had at least 12 months of natural amenorrhea.
7. Female subjects of child bearing potential with negative urine pregnancy tests on the day of screening and vaccination. Both male (if he has a partner of childbearing potential) and female subjects (of childbearing potential) must agree to use adequate and reliable contraceptive measures (e.g. spermicides, condoms, contraceptive pills) or practice abstinence for 10 days after vaccination.

#### Exclusion criteria

1. History of presence of cardiovascular, respiratory, hepatic, renal, gastrointestinal, neuropsychiatric, hematological, endocrine or immunosuppressive disorders that would be a risk factor when administered the study vaccines.
2. Positive serum anti-dengue IgG by enzyme-linked immunosorbent assay (ELISA).
3. Previous receipt of any yellow fever or Japanese encephalitis vaccines (including but not limited those that will be administered in this study).
4. Previous history of yellow fever or Japanese encephalitis infection.
5. Known allergy to JE-YF17D, YF17D or inactivated JEV vaccines or their components.
6. History of severe food/drug/vaccine allergies e.g. angioedema, anaphylaxis.
7. Known allergy to egg or egg products.
8. History of thymus gland disease.
9. Diagnosed with cancer or on treatment for cancer (with the exception of localized basal cell carcinoma) within 3 years prior to screening.
10. Evidence of clinically significant anemia (Hb <10 g/dl).
11. Blood donation exceeding >450mls in the past 3 months.
12. Presence of acute infection in the preceding 7 days or presence of a temperature ≥ 38.0°C (oral or tympanic temperature assessment), or acute symptoms greater than of “mild” severity on the scheduled date of first dose.
13. Pregnant or breast feeding women.
14. Evidence of substance abuse, or previous substance abuse including alcohol.
15. Participation in a study involving administration of an investigational compound (including investigational vaccines) within the past three months, or planned participation during the duration of this study.
16. Receipt of anti-inflammatory drugs (such as NSAIDs or systemic steroids) in the past 7 days.
17. Receipt of any licensed vaccine in the past 30 days before the first study vaccine dose.
18. Any condition that, in the opinion of the investigator, would complicate or compromise the study or wellbeing of the subject.

### Study design

56 subjects will be randomized into 1 of 2 arms (Arm 1 and Arm 2) in a 1:1 ratio. These two arms will be double-blinded. Subjects in Arm 1 will receive JE-YF17D vaccine (IMOJEV^®^, Sanofi Pasteur) on Day 0, followed by YF17D vaccine (STAMARIL^®^, Sanofi Pasteur) on Day 28. Subjects in Arm 2 will receive YF17D vaccine on Day 0 followed by JE-YF17D vaccine on Day 28. A separate non-randomized non-blinded cohort of 14 subjects will be recruited into Arm 3. Subjects in Arm 3 will receive inactivated JEV vaccine (IXIARO^®^, Valneva) on Day 0 followed by YF17D vaccine on Day 28. **Table 1** summarizes the study design.

**Table 1:** Study design.

Arms	Vaccination schedule	
	1^st^ vaccination (Day 0)	2^nd^ vaccination (Day 28)
Arm 1* (n=28)	JE-YF17D vaccine (IMOJEV^®^, Sanofi Pasteur)	YF17D vaccine (STAMARIL^®^, Sanofi Pasteur)
Arm 2* (n=28)	YF17D vaccine (STAMARIL^®^, Sanofi Pasteur)	JE-YF17D vaccine (IMOJEV^®^, Sanofi Pasteur)
Arm 3** (n=14)	Inactivated JEV vaccine (IXIARO^®^, Valneva)	YF17D vaccine (STAMARIL^®^, Sanofi Pasteur)

*Subjects (n=56) will be randomized in a double-blind manner to either Arms 1 or 2. **A separate cohort (n=14) will be recruited into Arm 3 in a non-randomized, single arm, open label design.

The rationale for these three study arms is as follows: Arm 1 will show the impact of a lower level of RNAemia induced by JE-YF17D vaccination (as compared to YF17D vaccination), and the resultant lower levels of virus-specific CD4+ and CD8+ T cells, would have on YF17D infection. In contrast, YF17D vaccination in Arm 2 would produce higher levels of RNAemia (as compared to JE-YF17D vaccination), and in turn higher levels of virus-specific T cells, thus likely resulting in greater suppression of JE-YF17D infection. The first vaccination in Arms 1 and 2 would provide the RNAemia response in the absence of virus-specific T cells, which would serve as a reference point to interpret the outcome of the second vaccination. For example, the level of suppression of JE-YF17D RNAemia will be the difference in RNAemia levels between subjects who received JE-YF17D as a second vaccination (Arm 2) compared to those who received it as a first vaccination (Arm 1), and similarly for YF17D vaccine. Arm 3 will serve as the control arm, as vaccination with inactivated JE vaccine should not produce any YF17D-specific T cell response.

### Randomization

For Arms 1 and 2, subjects will be randomized in a 1:1 ratio. 56 opaque sealed envelopes, each containing a study arm, will be randomly picked by a non-team member.

### Study visits and procedures

Informed written consent will be sought from subjects who fulfil criteria for enrolment. All consented subjects will undergo screening, which includes physical examination, full blood count, liver function test, and urinary pregnancy test (for female subjects of child-bearing potential). A flavivirus serostatus screen will also be performed using serum anti-dengue IgG as a surrogate marker for past flavivirus exposure.

**Table 2** shows the detailed study schedule. Study visits will occur on Day 0, 4, 7, 10, 14, 28, 29, 32, 35, 38, 42, 58. Subjects will receive vaccination on Day 0 and Day 28; at these visits blood sampling will be performed prior to vaccination. Subjects will undergo physical examination and research blood sampling at each study visit. Research blood samples will be collected at various time-points (see **Table 2**) for RNAemia measurement, T cell analysis, and antibody titer measurement. Urine samples will also be collected on Days 10, 14, 38 and 42 for urine viral RNA level measurement.

**Table 2:** Study schedule.

	Screening (D-14 to D-1)	D0	D4 (+1d)	D7	D10 (+/- 1d)	D14 (+/- 2d)	D28	D29	D32 (+1d)	D35	D38 (+/- 1d)	D42 (+/- 2d)	D58 (+/- 3d)
**Informed consent**	x												
**Eligibility check**	x	x											
**Medical history and demographics**	x												
**Physical examination**	x	x	x	x	x	x	x	x	x	x	x	x	x
**Vital signs** ^a^	x	x	x	x	x	x	x	x	x	x	x	x	x
**DENV IgG ELISA**	x												
**FBC, Liver panel** ^b^	x												
**Urine pregnancy test** ^c^	x	x					x						
**Randomization** ^e^		x											
**Vaccination**		x					x						
**RNAemia level (NS5 PCR)**		x^d^	x	x	x		x^d^		x	x	x		
**T-cell studies**		x^d^		x	x	x	x^d^		x	x	x	x	x
**PRNT**		x^d^					x^d^						x
**Anti NS1 antibody**		x^d^	x	x	x		x^d^		x	x	x		x
**Urine NS5 PCR**					x	x					x	x	
**Adverse event monitoring**		x	x	x	x	x	x	x	x	x	x	x	x
**Concomitant medication history**		x	x	x	x	x	x	x	x	x	x	x	x

a Vital signs include temperature (oral or tympanic), blood pressure, pulse rate, and respiratory rate.

b FBC includes haemoglobin, red blood cell count, white blood cell count, haematocrit, platelet count, neutrophil, lymphocyte, monocyte, eosinophil, basophil. Liver panel includes total protein, albumin, total bilirubin, alkaline phosphatase, alanine aminotransferase, aspartate aminotransferase.

c For female subjects of child bearing potential only.

d Blood sampling will be performed before vaccine administration on Day 0 and Day 28.

e Only applicable to Arms 1 and 2. Randomization will not be performed for Arm 3. DENV, dengue virus; IgG, immunoglobulin G; ELISA, enzyme linked immunosorbent assay; FBC, full blood count; PCR, polymerase chain reaction; PRNT, plaque reduction neutralization test.

e “x” indicates that the study procedure will be performed.

All subjects will be trained to observe for systemic vaccine-related symptoms (e.g. fever, myalgia, fatigue, headache) post-vaccination. A diary will also be given to the subjects to record such events should they occur during this period. Should they develop systemic symptoms that require intervention, they will report to the study site for medical evaluation and receive the appropriate therapy. Duration of symptoms will be recorded. Any concomitant medication use during this period will also be recorded.

### Study drug administration

As per manufacturer’s instructions, the administered dose of each reconstituted vaccine will be as follows:

1. YF17D (STAMARIL^®^, Sanofi Pasteur): 0.5 mls (4.0 - 5.8 log plaque forming units [PFU]) *via* subcutaneous injection
2. JE-YF17D (IMOJEV^®^, Sanofi Pasteur): 0.5 mls (3 - 4 log PFU) *via* subcutaneous injection
3. Inactivated JEV (IXIARO^®^, Valneva): 0.5 mls *via* intramuscular injection

In Arms 1 and 2, both JE-YF17D and YF17D vaccines will be administered by subcutaneous injection into the deltoid region of the left/right arm. The second (Day 28) vaccination will be delivered into the ipsilateral arm e.g. if the Day 0 vaccination is administered into the right arm, the Day 28 vaccination will also be administered into the right arm. In Arm 3, inactivated JEV will be administered by intramuscular injection into the deltoid region of the left/right arm and YF17D vaccine will be administered by subcutaneous injection into the deltoid region of the ipsilateral arm.

### Contraception and pregnancy testing

A urine pregnancy test will be performed at screening and on the day of vaccination (Day 0 and Day 28) for female subjects of child-bearing potential. Only those with a negative urine pregnancy test will be considered to be eligible for the study, provided that other eligibility criteria were fulfilled. Both male (if he has a partner of childbearing potential) and female subjects (of childbearing potential) must agree to use adequate and reliable contraceptive measures (e.g. spermicides, condoms, contraceptive pills) or practice abstinence for 10 days after vaccination.

### Biological specimen analysis

Biological specimens collected at time-points as shown in Table will be analyzed as follows:

#### T cell studies

The quantity of activated/proliferating T cells induced by vaccination will be assessed by measuring the frequency of activated effector CD4+ and CD8+ T cells expressing the activation markers CD38, and HLA-DR. The simultaneous expression of these two markers has been shown to accurately quantify the magnitude of T cell responses induced by live-attenuated viral vaccines (18, 19). Briefly, peripheral blood mononuclear cells (PBMCs) will be stained with a fixable live/dead marker and with antibodies specific to the surface markers CD3, CD4, CD8, HLA-DR and CD38. Stained cells will be fixed and then analysed by flow cytometry, as described previously (18).

The magnitude of YF17D-specific memory T cell responses induced by vaccination will be measured using three complementary methods: 1) Enzyme-linked immunospot assay (ELISpot), 2) Whole-blood cytokine release assay (CRA), and 3) Activation-induced Marker (AIM) assay. For all three assays, pools of 15-mer peptides overlapping by 10 amino-acids that span the C and NS1 to NS5 proteins of YF17D will be used to stimulate either PBMCs or whole blood *ex-vivo.* Overnight (16 hours) stimulation will be performed for the ELISpot assay (PBMCs) and CRA (whole blood), while a 24 hour stimulation will be performed for the AIM assay (PBMCs). ELISpot is a traditional assay which detects T cells that secrete IFN-γ in response to peptide stimulation. The whole-blood CRA measures the level of cytokines (e.g. INF- γ and IL-2) released by T cells in response to peptide stimulation and has shown good correlation with the ELISpot method (20). Finally, the AIM assay will be used to decipher whether the antigen-specific T cell response is sustained preferentially by CD4+ or CD8+ T cells – Briefly, PBMCs collected at various time points will stained with antibodies to the surface markers CD3, CD4, CD8 and activation markers CD134, CD137 and CD139. Stained cells will be fixed and then analysed by flow cytometry, as described previously (21).

#### Vaccine RNAemia

Vaccine RNAemia (as well as urine viral RNA levels) will be measured using RT-qPCR directed against the NS5 gene of YF17D, allowing RNAemia levels induced by JE-YF17D vaccination and YF17D vaccination to be directly compared.

#### Neutralizing antibody titers

Neutralizing antibody titers to YF17D and JE-YF17D will be measured by plaque reduction neutralization test (PRNT) as described previously (16, 22). Briefly, 40 pfu of YF17D or JE-YF17D virus will be reacted with serial two-fold diluted serum samples and incubated at 37°C. This mixture will then be added to a monolayer of BHK cells and incubated for one hour before the addition of maintenance medium with carboxymethylcellulose. Back titration of virus will also be carried out to ensure that the amount of virus used is as indicated. The virus and cells will then be incubated for 3-5 days, following which, the cells will be washed, fixed and stained with crystal violet. The number of plaques will then be counted and PRNT_50_ titers estimated.

### Study discontinuation and withdrawal

Subjects are free to withdraw consent and discontinue their participation at any time without prejudice to them or effect on their medical care. Subjects may be withdrawn, if necessary, to protect their health or the integrity of the study. The investigator also has the right to withdraw participants from the study in the event of inter-current illness, adverse events, treatment failures, protocol violations, administrative, or other reasons. When a subject withdraws from the study, no new data will be collected for study purposes unless the data concern an adverse event related to the study. All details available will be reported and recorded for any subject that withdraws or is removed from the study. Subjects who drop-out or withdraw from the study will not be replaced.

The Principal Investigator (PI) may stop a subject’s participation in the study at any time for one or more of the following reasons: Failure to follow the instructions of the PI and/or study staff, the PI decides that continuing participation could be harmful to the subject, pregnancy, the study is cancelled, and/or other unanticipated circumstances.

## Data collection and management

### Data entry and storage

Direct data capture of demographic and clinical data will be conducted using computer notebooks. The data will be kept confidential in password protected computers accessible only by delegated research staff. Identifiers will be kept in a separate file in another office and every effort will be made to protect the privacy of the participants. The data to be analyzed will contain only de-identified data. An electronic data capture system (RedCap) will be used.

### Data quality assurance

The PI will review the study periodically for data and safety monitoring. Internal quality checks will be performed by two Clinical Research Coordinators (CRCs) who are study team members. The data entered by one CRC will be checked by another using the source documents. The study may also be evaluated by government inspectors/regulatory authorities who must be allowed access to the electronic data capture system (RedCap), source documents, and other study files. The inspectors will review CRFs and compare them with source documents to verify accurate and complete collection of data and confirm that the study is being conducted according to the protocol, ICH-Good Clinical Practices and all applicable regulations.

### Sample size calculation

To detect an effect size of 0.8 SD in mean RNAemia level on log (10) scale between the first and second dose with the same vaccine – first dose of YF17D will be analysed against those who received YF17D after JE-YF17D and vice versa – a sample size of 25 per group will provide 80% power at a 5% two-sided type 1 error rate. To allow for a drop-out rate of 10%, a total sample size of 28 per group for Arms 1 and 2 is targeted. For Arm 3, which is the control group, a sample size of 14 will be used.

### Data and statistical analysis

Distributional diagnostic plots will be used to examine the shape of distributions of T-cell counts and RNAemia levels for each of the vaccines. Parametric or non-parametric procedures will be used to assess the differences in RNAemia levels T-cell counts, antibody titers and cytokine levels, as appropriate. The peak frequencies of activated CD4+ and CD8+ T cells will be compared using the student’s t-test. The duration of activated CD4+ and CD8+ T cell responses will be compared using area the curve (AUC), and the differences will be assessed using the Wilcoxon-rank-sum test. The level of antigen-specific cytokine production will be compared within each vaccine. Overall kinetics of cytokine production will be assessed for each time-point and compared with the baseline levels using a paired t-test. The peak cytokine production and AUC will be compared between both vaccines using the student’s t-test or Wilcoxon-rank-sum test, depending on whether the distribution is parametric or non-parametric, using the Shapiro-Wilk normality test or equivalent. The relationship between the activated T-cell frequencies and cytokine levels will be assessed using Spearman’s correlation analysis. To compare differences in RNAemia, both peak RNAemia and AUC will be compared using the student’s t-test or Wilcoxon-rank-sum test, depending on whether the distribution is parametric or non-parametric, using the Shapiro-Wilk normality test or equivalent, respectively.

The frequency and type of adverse events reported will first be analysed by determining the median and the interquartile ranges. Differences in frequencies of adverse events between the first and challenge vaccination will be analysed using 2x2 tables and chi square analysis or Fisher’s exact test, whichever is appropriate. Parametric or non-parametric procedures will be used to assess the differences in pre-challenge virus-specific T-cell counts among those with adverse events and those without.

Linear regression analysis between peak RNAemia and T cell responses will performed to determine the minimal level of T-cell responses needed to reduce peak RNAemia by 1 log genome copies/ml. If, however, the correlation is non-linear (p > 0.05) a polynomial fit will be used to evaluate if a good model fit can be attained. Finally, in the unlikely event that none of these models fit the data points, the T-cell response for all subjects will be consolidated, and the T-cell responses that are at least 3 standard deviations away from the mean T-cell responses will be determined. These data points will be labelled as outlier responses, and the corresponding level of fold-change reduction in RNAemia will be determined. For all statistical comparisons, a p-value of <0.05 will be considered significant.

## Safety assessment

### Definitions

An adverse event (AE) is any untoward medical occurrence in a patient or clinical investigation subject administered a pharmaceutical product and which does not necessarily have a causal relationship with this treatment. An AE can therefore be any unfavorable and unintended sign (including an abnormal laboratory finding), symptom, or disease temporally associated with the use of a medicinal (investigational) product, whether or not related to the medicinal (investigational) product.

A serious adverse event (SAE) is any untoward medical occurrence that at any dose results in death, is life-threatening, requires inpatient hospitalization or prolongation of existing hospitalization, results in persistent or significant disability/incapacity, or is a congenital anomaly/birth defect.

AEs will be graded according to the Common Terminology Criteria for Adverse Event Monitoring, version 5.0. Event terminology, the date and time of event start and end, severity, relatedness, impact to the continuation of the study, and final outcome of the event will be recorded on the case report form from day 0 to the last study visit until the event has been resolved. For a SAE, in addition to the above, an event summary, the criteria used to categorize the event as an SAE, and a list of all tests and treatments given for the event will be documented.

### Serious adverse event reporting

SAE reporting requirements will be in accordance with the SingHealth CIRB protocols. Only related SAEs (definitely/probably/possibly) will be reported to CIRB. Related means there is a reasonable possibility that the event may have been caused by participation in the clinical trial. The PI will be responsible for informing CIRB after first knowledge that the case qualifies for reporting. Follow-up information will be actively sought and submitted as it becomes available. Related AEs (non-SAE) will not be reported to CIRB. However, the investigator is responsible to keep record of such AEs cases at the Study Site File.

All SAEs that are unexpected and related to the study drug will be reported to Health Sciences Authority (HSA) of Singapore. The PI is responsible for informing HSA no later than 15 calendar days after first knowledge that the case qualifies for expedited reporting. Follow-up information will be actively sought and submitted as it becomes available. For fatal or life-threatening cases, HSA will be notified as soon as possible but no later than 7 calendar days after first knowledge that a case qualifies, followed by a complete report within 8 additional calendar days.

### Study monitoring

The study may be evaluated by government inspectors/regulatory authorities who must be allowed access to electronic case report forms (CRFs), source documents, and other study files. The inspectors will review CRFs and compare them with source documents to verify accurate and complete collection of data and confirm that the study is being conducted according to the protocol, ICH-Good Clinical Practices and all applicable regulations.

## Discussion

Although this study is focused on flaviviral vaccines, T cell immunity also plays a critical role in other viruses and viral vaccines. For example, although current COVID-19 mRNA vaccines are not able to sustainably protect against infection by the Omicron variant due to antibody escape mutations, protection against severe disease is maintained through preserved virus-specific T cell responses (23–28). We have also previously shown that the induction of T cell responses coincide with the onset of COVID-19 vaccine-induced protection, even prior to the appearance of neutralizing antibody responses (29). Thus, the findings from this trial would be applicable to many viral diseases.

The experimental medicine approach will enable the systematic study of the host T cell immune response to both vaccination and acute viral infection in a safe, controlled and ethical manner. In contrast, a “real world” study involving patients naturally infected with wild-type virus would be confounded by multiple factors, including virus strain variations and size of virus inoculum. As the exact timing of infection would be unknown, such studies would also encounter difficulty in obtaining pre- and post-infection samples at fixed time-points, and would require an unfeasibly large sample size in order to successfully test our hypotheses.

It can be argued that this study makes use of attenuated viruses, and thus the magnitude and quality of protective T cell responses measured would underestimate the threshold needed for immunity against wild-type virus as well as non-flaviviruses. Although this argument holds, our goal, however, is not to define a universal T cell correlate of protection. Rather, we submit that the use of attenuated viruses would establish a minimum threshold – the quantity and quality of T cell responses below such a threshold would unlikely be able to control any systemic wild-type viral infection. Such information would be foundational when selecting promising vaccine candidates for further clinical development.

## Ethics statement

The studies involving human participants were reviewed and approved by SingHealth Centralised Institutional Review Board. The patients/participants provided their written informed consent to participate in this study.

## Author contributions

EEO, JL, SK, KC, OS, EZO, and AB conceived of and designed the study. EEO obtained the funding. SK, YC, and JL set up the recruitment sites. SK wrote the first draft of the manuscript. All authors participated in the critical review, revision and approval of the manuscript. All authors contributed to the article and approved the submitted version.
